# Crizotinib in Sarcomatous Malignancies Harboring ALK Fusion With a Definitive Partner(s): Response and Efficacy

**DOI:** 10.3389/fonc.2021.684865

**Published:** 2021-10-14

**Authors:** Jinchun Wu, Yongbin Hu, Omar Abdihamid, Gengwen Huang, Sheng Xiao, Bin Li

**Affiliations:** ^1^ Department of Oncology, Xiangya Hospital, Central South University, Changsha, China; ^2^ Department of Pathology, Xiangya Hospital, Central South University, Changsha, China; ^3^ Department of Pancreatic Surgery, Xiangya Hospital, Central South University, Changsha, China; ^4^ Department of Pathology, Brigham and Women’s Hospital, Harvard Medical School, Boston, MA, United States

**Keywords:** crizotinib, sarcoma, sarcomatoid malignancies, ALK fusions, definitive partner(s), adverse events

## Abstract

Sarcoma or sarcomatoid malignancies are a set of mesenchymal-origin malignancies with vast heterogeneity in clinical and molecular characteristics. Anaplastic lymphoma kinase (ALK) is a tyrosine kinase oncoprotein expressed by several tumors, including sarcomas. Crizotinib is an effective ALK inhibitor. In this review paper, we summarized findings from the literature regarding the use of crizotinib for the treatment of sarcoma and sarcomatoid malignancies harboring ALK fusions with definitive partners (with the given gene(s) name) from the years 2010 to 2021.One hundred and four articles were retrieved and after exclusion, 28 studies containing 33 patients were finally selected. All 33 patients were treated with crizotinib. Among the 33 cases, 19 were adult patients, 11 were pediatric patients, and 3 cases did not have data on age and/or gender. Most cases had a primary abdominal lesion (16/30), followed by thoracic (10/30), trunk (3/30), retroperitoneal (1/30), and one case of right medial thigh (case 7). Stage IV disease was reported in 76.7% (23/30) of patients. The objective response rate and disease control rate was 86.7% (26/30) and 96.7% (29/30), respectively, which were assessed on average of 8 weeks after crizotinib initiation. Rapid improvement of symptoms was observed within one to two weeks in some cases including patients with extensive diseases or poor performance. There was no difference in crizotinib response between pediatrics and adult cases. Crizotinib is effective; however, surgery remains the mainstay of therapy, with newer evidence showing concurrent crizotinib with surgery conferring long-term overall survival. However, we should still be cognizant of the heterogeneous landscape of crizotinib efficacy and its associated fatal adverse events.

## Introduction

Anaplastic lymphoma kinase (ALK), a single-chain transmembrane receptor tyrosine kinase, was first described as a fusion partner in the t (2;5) chromosomal translocation in anaplastic large cell lymphoma (ALCL) in 1994. The C-terminal 563 amino acids (aa 1058-1620) that constitute the cytoplasmic portion of ALK, including the kinase domain, are encoded by exons 20-29 at the 3’ end of the ALK gene. The resulting fusion oncoproteins are chimeric self-associating polypeptides with a variety of N-terminal domains and a common, constitutively active C-terminal tyrosine kinase domain, in the vast majority of cases including all 563 cytoplasmic amino acids from ALK. Novel ALK fusion protein partners are increasingly uncovered in sarcoma or sarcomatoid carcinoma (1–3). Endoplasmic reticulum ribosomal-binding protein 1 (RRBP 1), Glypican1 (GPC1), leucine-rich repeat flightless-1-interacting protein 1 (LRRFIP1), Fibronectin 1 (FN1), or Tensins1 (TNS1), and Nuclear Mitotic Apparatus proteins (NUMA1), were correlated with carcinogenesis and cancer progression *via* multifaceted methods such as reducing ER stress, as modulators of growth factor signaling, promoting epithelial mesenchymal transition (EMT), participating in cellular adhesion and migration processes, or be involved in deoxyribonucleic acid (DNA) damage repair and homologous recombination et al. So different ALK fusion proteins exhibit divergent stability, cellular distribution and sensitivity to ALK tyrosine kinase inhibitors(TKIs) due to distinct characteristics of the partners which were fused to ALK gene.

Crizotinib is a kinase inhibitor approved by Food and Drug Administration (FDA) for the treatment of ALK or c-ros oncogene 1 receptor kinase (ROS1) -positive patients with metastatic non-small cell lung cancer (NSCLC). It was gradually off-label in the treatment of inoperable sarcomas that are carried with ALK fusions since the first case of inflammatory myofibroblastic sarcoma (IMT) with the ALK rearrangement showed a favorable response to crizotinib (4). However, several questions remain unanswered, including if single-agent crizotinib is optimal, or if a partner of ALK fusion kinase variants influences tumor response to crizotinib (5), and if next-generation ALK inhibitors are effective post-crizotinib resistance.

Owing to the low incidence of *ALK-*fusion sarcoma and sarcomatoid malignancies, most of the available data are case reports with detailed molecular profiles. Therefore, we aim to provide a systematic review of the available data on the therapeutic effects of crizotinib in ALK-positive sarcoma and sarcomatoid malignancies.

## Materials And Methods

### Medical Subject Heading Terms (MESH)

An electronic literature search in the PUBMED database was performed on August 22^nd^, 2021. The search period was from 2010 to 2021. MESH terms used for the search were “ ALK*[AB] AND [CRIZOTINIB*(AB) OR ALK inhibitor (TI) OR kinase inhibitor (TI)] AND [SARCOMA*(AB) OR SARCOMATOID(TI) OR INFLAMMATORY MYOFIBROBLASTIC TUMOR (TI)]”. All articles included in this review were identified according to the inclusion criteria. Conference abstracts were excluded.

### Eligibility Criteria

All articles were screened as per inclusion criteria: All patients with sarcoma or sarcomatous malignancies, presence of *ALK* fusion with definitive partner(s) and use of crizotinib. The exclusion criteria include non-human models, non-English language publications, and reviews that do not contain original case reports.

### Data Collection

All included articles were reviewed, and the following information was retrieved: Year of publication, partners for ALK fusions, tumor histomorphology, patient age and gender, sequence of crizotinib use and its combination, treatment response, survival time, and adverse events (AEs).

### Statistical Methods for Pooled Analyses

Pooled analyses were performed using SPSS software, in which normally distributed data were represented as average (95%CI, lower and upper limit). Data that were not normally distributed were presented as medians (95% CI, lower and upper limit). Chi-square test was used to compare the response of crizotinib between the different subgroups. Survival was analyzed using the Kaplan-Meier curves. P value less than 0.05 was considered statistically significant.

## Results

### Study Selection

**Figure 1:**
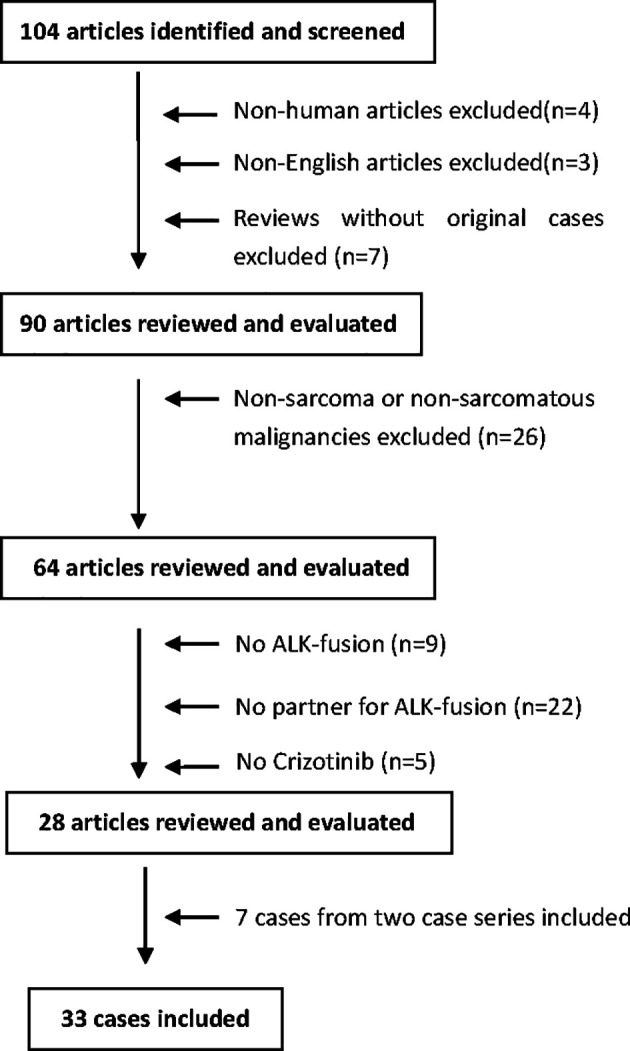
Flow chart of the literature screening in this study.

As shown in **Figure 1**, a total of 104 search results were found. After screening, the following articles were excluded: non-human data (n=4), non-English language articles (n=3), reviews without original cases (n=7), non-sarcoma/non-sarcomatous malignancies (n=26), lack of ALK-fusion (n=9), lack of ALK-fusion partners (n=22), and lack of crizotinib-based drug regimens (n=5). In addition, 7 cases from two case series (n=7) were presented individually and included to generate a total of 33 cases (26 + 7) that were analyzed in this review.

### Patients and Tumors Characteristics

Among the 33 cases in **Table 1**, 19 were adult patients, 11 were pediatric patients, and 3 cases did not have data on age and/or gender. Most cases had a primary abdominal lesion (16/30), followed by thoracic (10/30), trunk (3/30), retroperitoneal (1/30), and one case of right medial thigh (case 7). Stage IV disease was reported in 76.7% (23/30) of patients.

**Table 1:** Crizotinib treatment in ALK-fusion sarcoma and sarcomatous malignancies.

Case	Age(Y) /Gender	Histology	Staging/main lesions^!^	Partner/detection method(s)	Line/Best response (Crizotinib)/TTE	Combined Treatment/Response	Duration on/off Crizotinib	Additional therapy(response)after Crizotinib	Survival time after initial Crizotinib	Current status	Year Ref
**1**	44/M	EIMT	IV/Abdomen	RANBP2/RT-PCR and sequencing	3^rd^/PR/1.9ms	Surgery/CR	30ms/-	–	30ms	NED	2010 (4)
**2**	22/M	EIMT	IV/Abdomen	RANBP2/RT-PCR and sequencing	4^th^/PR/5ms	–	14ms/-	–	14ms	AWD	2014 (6)
**3**	22/M	EIMT	IV/Abdomen	RANBP2/RT-PCR and sequencing	4^th^/PR/1ms	–	10ms/-	–	10ms	AWD	2014 (7)
**4**	24/M	IMT	IV/Abdomen	CLTC/RT-PCR and sequencing	1^st^/SD/4ms	–	4ms/-	–	4ms	AWD	2014 (8)
**5**	50/F	STUMP	IV/Abdomen	DCTN1/NGS	3^rd^/NA	Pazopanib/PR/2cycles	6ms/-	–	6ms	AWD	2015 (9)
**6^#^**	8/F	IMT	IV/Thoracic	TPM3/Cytogenetics	2^nd^/PR/2ms	–	4ms/-	–	4ms	DOC/4ms	2016 (10)
**7**	32/M	IMT	IV/Thoracic	TPM3/MPSeq	2^nd^/PR/NA	–	8ms/PD	Celecoxib (PD) /Ceritinib(PR)/ Surgery, ablation	27ms	PD	2016 (11)
**8**	16/F	IMT	IV/Trunk	EML4/FISH	5^th^/PR/a few weeks	Surgery,LR/CR	36ms/-		36ms	NED	2016 (12)
**9**	28/M	EIMT	IV/Abdomen	RANBP2/NGS	1^st^/PR/2ms	–	9ms/PD	Brigatinib(PR)	28ms	AWD	2019 (13)
**10^**	17/M	EIMT	NA	RANBP2/NA	1^st^/Adjuvant/NA	–	NA/NA	Alectinib (Adjuvant)	NA	NED	2020 (14)
**11**	NA/M	Sarcoma	IV/Trunk	CARS/WGS	3^rd^/PR/4ms	Surgery/CR	41/7ms	Surgery (CR)Alectinib (Adjuvant)	48ms	NED	2020 (15)
**12**	22/M	EIMT	IV/Abdomen	RANBP2/FISH	1^st^/Adjuvant/NA	–	16ms/-	–	16ms	NED	2015 (16)
**13**	61/M	PSC	IV/Thoracic	EML4/ARMS-PCR	2^nd^/PD/0.5ms	–	0.5ms	–	0.5ms	DOD	2019 (17)
**14**	37/F	IMT	IV/Retroperitoneal	*Partners/FISH and RNA sequencing	2^nd^/CR/5ms	–	5/4ms	Surgery (CR)	9ms	NED	2019 (18)
**15^~^**	45/M	EIMT	IV/Abdomen	EML4/RT-PCR and sequencing	1^st^/PR/0.25ms	–	0.5ms/-	–	0.5ms	DOC/0.5ms	2017 (19)
**16**	15/F	EIMT	IV/Abdomen	RANBP2/NGS	5^th^/PR/0.75ms	–	0.75ms/intolerable	Ceritinib (NA)	24ms	AWD	2017 (20)
**17**	NA	MUO	NA	EML4/NGS	3^rd^/PR/NA	–	NA	–	NA	NA	2019 (21)
**18**	50/M	PSC	IV/Thoracic	EML4/NGS	2^nd^/PR/3ms	–	22ms/-	–	22ms	AWD	2018 (22)
**19**	0.7/F	IMT	IIIB/Abdomen	SEC31A/RNA sequencing	NA/SD/NA	–	10.8/44.4ms	–	55.2ms	AWD	2019 (23)
**20**	14.7/M	EIMT	IV/Abdomen	RANBP2/RNA sequencing	NA/CR/NA	–	4.8ms/Relpase	Ceritinib (CR)	NA	NED	2019 (23)
**21**	7.4/F	IMT	IV/Thoracic	CLTC/RNA sequencing	NA/PR/NA	Surgery/CR	20.4/30ms	–	50.4ms	NED	2019 (23)
**22**	11.3/M	EIMT	IIIB/Abdomen	RANBP2/RNA sequencing	NA/PR/NA	Surgery/CR	36/12ms	–	48ms	NED	2019 (23)
**23**	9.1/M	EIMT	IV/Abdomen	RANBP2/RNA sequencing	NA/CR/NA	–	12ms/Relapse	Ceritinib, chemo-, NSAIDs (PD)	22.6ms	DOD	2019 (23)
**24**	44/F	HS	IV/Thoracic	CLTC/NGS	2^nd^/PR/NA	–	4.4ms/PD	Alectinib(PD),Ceritinib(PR), Entrectinib(PD)	18.4ms	DOD	2021 (24)
**25**	NA	MS	NA	DCTN1/NA	1^st^/PR/1.5ms	Pazopanib	NA	NA	NA	NA	2021 (25)
**26**	6/F	IMT	T2NxMxGx/Thoracic	TRAF3/NGS	1^st^/CR/8ms	–	8ms/-	–	8ms	NED	2021 (26)
**27**	15/M	IMT	T4N_X_M_X_G_X_/Trunk	LRRFIP1/NGS	1^st^/PR/5ms	–	NA	–	NA	AWD	2020 (27)
**28**	43/F	IMT	T_X_N_X_M_X_G_X_/Abdomen	FN1/RNA-based fusion Plex sarcoma NGS	1^st^/SD/2ms	–	4ms/SD	Lorlatinib(PR)Surgery (CR)	17.3ms	NED	2020 (28)
**29**	21/F	IMT	IV/Thoracic	NUMA1/NGS	1^st^/PR/1ms	Nivolumab	6/7ms	Alectinib(PR)	13ms	AWD	2017 (29)
**30**	22/M	IMT	T4N_X_M_X_G_X_/Abdomen	RRBP1/WES and RNA sequencing	1^st^/PR/3ms	–	5.3ms/PD	Alectinib(SD), Surgery, PD, Ceritinib(PR)	31.5ms	AWD	2020 (30)
**31**	60/F	IMT	IV/Abdomen	TNS1/RNA sequencing	2^nd^/PR/1ms	–	3ms/PD	Alectinib (SD)	11ms	DOD	2020 (30)
**32^¥^**	61/M	IMT	IV/Thoracic	TPM3/NGS	1^st^/CR/NA	surgery	17/4ms	Alectinib(CR)	21ms	NED	2021 (31)
**33**	36/F	IMT	NA/Thoracic	DCTN1/Ion AmpliSeq	1^st^/PR/NA	–	29ms/PD	Ceritinib(PR)	40ms	DOD	2021 (32)

^!^According to American Joint Committee (AJCC) staging system.

^#^Acute lymphoblastic leukemia with hematopoietic stem cell transplant (HSCT). Crizotinib-related renal cyst, change to alectinib. ^$^Symptoms relief. *Partners-ALK: TPM3-ALK, MPRIP-ALK, KLC1-ALK, KIF5B-ALK, EML4-ALK, HIP1-ALK. ^~^Special administration: Crizotinib capsule was opened, and the powder was mixed with pure water. The mixture was then injected into the nasogastric tube, placed into the terminal ileum with a length of 50 cm from the stoma. ^￥^IMT located in the right intermediated bronchial trunk, with a simultaneous diagnosis of lung adenocarcinoma harboring EML4-ALK fusion, which was resistant to crizotinib after 17ms of treatment and was changed to alectinib. Y, year-old; M, male; F, female; EIMT, Epithelioid inflammatory myofibroblastic sarcoma; IMT, Inflammatory myofibroblastic sarcoma; STUMP, Smooth muscle tumor of uncertain malignant potential; PSC, Pulmonary sarcomatoid carcinoma; MUO, Malignancy of undefined origin; HS, Histiocytic sarcoma; MS, Myxoid sarcoma; TTE, Time to evaluation; NGS, Next-generation sequencing (DNA); MPSeq, Mate Pair sequencing; FISH, Fluorescence in situ hybridization; WGS, Whole-genome sequencing; ARMS-PCR, Amplification refractory mutation system-polymerase chain reaction; WES, Whole-exome sequencing; LR, Local radiation; NA, Not available; RD, Residual disease; SD, Stable disease; PD, Progressive disease; CR, Complete remission; PR, Partial remission; Ms, Months; NED, No evidence of disease; AWD, Alive with the disease; DOC, Die of complications; DOD, Dead of disease.

The histomorphology included 11 cases of epithelioid inflammatory myofibroblastic sarcoma (EIMT), 15 cases of IMT, 2 cases of pulmonary sarcomatoid carcinoma (PSC), 1 case of low-grade sarcoma, 1 case of smooth muscle tumor of uncertain malignant potential (STUMP), 1 malignancy with unknown origin, 1 histiocytic sarcoma (HS), and 1 myxoid sarcoma (MS).

Generally, most EIMTs harbor *RANBP2-ALK* (10/11) rearrangement. Using newer Ribonucleic Acid (RNA) sequencing technologies, the 15 cases of IMT were found to harbor various fusion partners of *ALK*, including *CLTC, TPM3, EML4, SEC31A, TRAF3, LRRFIP1, FN1, NUMA1, RRBP1, TNS1*, and *DCTN1*. Both cases of PSC harbored EML4-ALK. Similar to IMT, HS and MS harbored partners of CLTC and DCTN1. Intriguingly, one case report by Ogata et al. showed multiple fusion partners, including *TPM3* (tropomyosin 3), *MPRIP* (myosin phosphatase Rho interacting protein), *KLC1* (kinesin light chain 1), *KIF5B* (kinesin family member 5B), *EML4* (echinoderm microtubule-associated protein-like 4), and *HIP1* (huntingtin interacting protein 1).

### Response to Crizotinib Treatment

As shown in **Table 1**, the objective response rate (ORR) and disease control rate (DCR) were 86.7% (26/30) and 96.7% (29/30), respectively, which were assessed on average of 8 weeks after crizotinib initiation. One case with concurrent KRAS exon 2 mutation deteriorated clinically and died  (17). Rapid improvement of symptoms was observed within one to two weeks in some cases (case 3, 15, 29). Patients with extensive diseases or poor performance status (PS) (case 6, 15, 29, 30) still had good responses to crizotinib. Intriguingly, EIMT had an excellent ORR of 100% to crizotinib (9/9). One IMT case with FN1 partner mutation had no response to crizotinib, but the tumor dramatically shrunk following lorlatinib neoadjuvant treatment followed by radical resection (28). Another IMT case with a TNS1 partner progressed shortly after the crizotinib response and died upon switching to alectinib (30). Three pediatric cases (cases 21, 22, 19) were off crizotinib upon achieving no evidence of disease (NED) or stable disease (SD), with recurrent lesions surgically removed, hence achieving long-term survival  (23). There was no difference in crizotinib response between pediatric and adult cases (P=0.809) (**Table 2**).

**Table 2:** Response to crizotinib treatment in pediatric and adult patients.

Age/response	Pediatrics	Adults	P-value
CR	3	2	
PR	6	12	
SD	1	2	
PD	0	1	
Total	10	17	0.809

Age, pediatrics (<18 yrs.); adults; (≥18 yrs.); CR, complete remission; PR, partial remission; SD, stable disease; PD, progressive disease.

p value was generated using the Chi-square test.

### Improved Outcomes in Patients Treated With Surgery and Crizotinib

**Figure 2:**
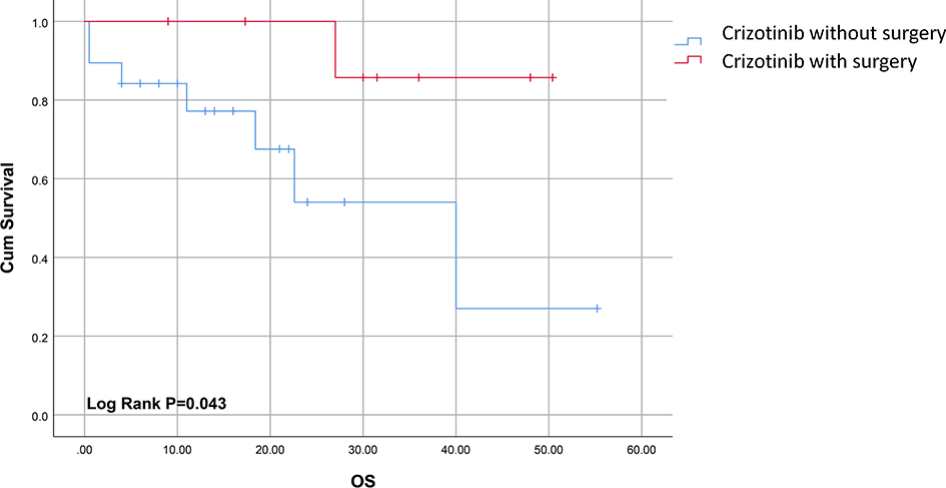
Survival curves of ALK-positive sarcomatous malignancies for patients treated with crizotinib with or without surgery.

The survival time for cases included in our analysis ranged from 0.5 to 55.2 months. Patients treated with both surgery and crizotinib had an improved clinical outcome compared to cases treated with crizotinib alone as indicated by complete remission (CR) rates of 80% (8/10) and 5.3% (1/19), respectively (P<0.05) (**Table 3**). The median overall survival (mOS) time in the combination group was significantly longer compared to crizotinib treatment alone (31.5ms, 95% CI, 17.3-48.0ms; versus 14ms, 95% CI, 8.0-22.0ms, respectively, P<0.05) (**Figure 2**). Gaudichon et al. reported a metastatic IMT case that achieved CR following multiple primary lesion resection and a metastasectomy, all while on crizotinib maintenance therapy (12).

**Table 3:** Response to crizotinib therapy with or without surgery in patients with sarcomatous malignancies.

Treatment/response	Crizotinib without surgery	Crizotinib with surgery	P-value
CR	1	8	
PR	10	0	
SD	4	0	
PD	4	2	
Total	19	10	<0.05*

CR, complete remission; PR, partial remission; SD, stable disease; PD, progressive disease.

p value was generated using the Chi-square test. *Indicates statistical significance at p < 0.05.

### AEs of Crizotinib

As shown in **Table 4**, crizotinib-related AEs were observed in 16 cases, with some cases having multiple AEs  (4, 7, 13, 18, 22). Serious AEs, including transaminitis, neutropenia, renal cyst, cellulitis, and multiple fractures, were seen in 7 cases prompting crizotinib cessation or dose reduction (1, 7, 18, 20, 23, 29, 30). Fatal AEs in 2 cases led to death, which occurred in 0.5 and 4ms, respectively, after crizotinib initiation. One case was an undiagnosed tumor lysis syndrome (TLS), in which a heavy tumor burden caused a rapid fatal response to crizotinib, while the other was due to criztoinib-related acute respiratory distress syndrome (ARDS) (10, 19). Notably, one case achieved partial remission (PR) following combination therapy with crizotinib and nivolumab due to high PD-L1 expression in tumor tissues but later presented with recurrent pneumonitis resulting from both agents prompting a switch to alectinib  (29).

**Table 4:** Crizotinib-related AEs in ALK-fusion sarcomatous malignancies.

	Consequence	Frequencies ^Ref^
**AEs** **(Grade 1-2)**		**7 cases**
Extremities edema	–	2 (4, 13)
Joint aches	–	1 (4)
Hypocalcemia	–	1 (4)
Hypophosphatemia	–	1 (4)
Myelosuppression	–	2 (4, 13)
Fatigue	–	1 (7)
Nausea	–	2 (7, 12)
Dizziness	–	1 (7)
Diarrhea	–	1 (18)
Visual disturbance	–	2 (7, 18)
Ear ringing	–	1 (7)
Dysgeusia and itching	–	1 (7)
A light cough	–	1 (22)
Lower-abdominal cramping		1 (29)
ALT/AST elevation	–	1 (22)
**AEs** **(Grade 3-4)**		**7 cases**
ALT/AST elevation	Crizotinib withheld	2 (7, 18)
Intolerable toxicity	Crizotinib cessation	1 (20)
Neutropenia	Crizotinib dose reduction or cessation	2 (23, 24)
Renal cyst	Crizotinib cessation	1 (14)
Cellulitis	Crizotinib cessation	1 (23)
Multiple fractures	Crizotinib cessation	1 (23)
Pneumonitis	Crizotinib withheld	1 (29)
**Fatal AEs**		**2 cases**
ARDS	Death	1 (10)
TLS	Death	1 (19)

ALT, alanine transaminase; AST, Aspartate transaminase; ARDS, acute respiratory distress syndrome; TLS, tumor lysis syndrome.

### Post-Crizotinib Treatment Options and Outcomes

Ten cases had no response or progressed on crizotinib treatment (11, 13, 15, 23, 24, 28, 30, 32). Nine of them received next-generation ALK inhibitors (ceritinib, brigatinib, alectinib, or lorlatinib) with 77.8% (7/9) achieving ORR (11, 13, 23, 24, 28, 30, 32), and the remaining two cases resistant to ceritinib or alectinib died within one year after their progression on crizotinib (23, 30). One case with radical surgery followed by alectinib adjuvant treatment was still alive without evidence of disease (15). Two cases showed no response to alectinib but achieved tumor shrinkage following subsequent ceritinib treatment  (24, 30).

## Discussion

ALK protein is normally expressed in the central nervous system and encodes a novel receptor, tyrosine kinase (33). ALK rearrangement leads to its dysregulation under a new promoter from its fusion partner genes, resulting in ligand-independent tyrosine kinase activation. ALK fusions were identified in about 50% of IMT cases, 3.6% of sarcomatous malignancies, and extremely rare in rhabdomyosarcomas and leiomyosarcoma  (34).

ALK-positive sarcoma is predominantly seen in pediatric or middle-aged patients, with only 2 patients older than 60-years in our cohort of 33 cases. IMT and its variant EIMTs composed the majority of our cases, accounting for 78.8% (26/33). Crizotinib is an effective Adenosine triphosphate (ATP)-competitive ALK inhibitor. It achieved 74% ORR in ALK-fusion positive NSCLC (35). But the efficacy of crizotinib in sarcoma or sarcomatous malignancies is largely unknown. Although limited by sample size, our pooled analysis showed up to 86.7% efficacy of crizotinib in ALK-positive sarcoma or sarcomatoid malignancies with equal effectiveness in both adult and pediatric patients.

The partners of ALK fusion kinase variants impact cellular phenotypes and response to ALK inhibitors  (5). As shown in case 28, FN1 is ubiquitously and abundantly expressed in human cells; therefore, high-level expression of ALK under the control of a strong FN1 promoter, as revealed by immunohistochemistry, leads to reduced crizotinib efficacy  (36). TNS1-ALK fusion in case 31 also represents a distinct variant of crizotinib resistance but was later sensitive to brigatinib, which controlled disease for at least 9 months  (37). Similar to previous reports, most EIMTs have RANBP2-ALK mutation. RANBP2 is a perinuclear protein closely related to mitochondrial stress, a feature that likely explains distinct staining patterns around the perinuclear and nuclear membrane and the aggressive nature of EIMTs  (38). However, RANBP2 does not affect crizotinib response; as shown in **Table 1**, all EIMT cases responded to crizotinib.

Surgical resection remains the mainstay of treatment for localized ALK-positive sarcoma and sarcomatous malignancies. Crizotinib combined with surgery improved long-term survival, with mOS of up to 31.5ms, compared to crizotinib alone(mOS 14ms). Resection of resistant or recurrent lesions such as oligo-residual or oligo-progression is optimal for treating sarcomas  (39). A main example of this approach is the role of surgery in prolonging the progression-free survival (PFS) after imatinib resistance in gastrointestinal stromal tumors (GIST), mainly by resection of resistant tumor lesions  (40).

Mutations within the ALK fusion gene attribute to about 30% of cases of crizotinib resistance. Second generation of ALK TKIs, including ceritinib, brigatinib, alectinib, were usually effective in later lines (13, 32), similar to their current indications in NSCLC. Interestingly, 2 cases that progressed on alectinib following crizotinib resistance showed response to subsequent ceritinib (24, 30). Different ALK inhibitors bind slightly differently within the ATP-binding pocket of the ALK kinase domain, and thus coax different resistance mutation patterns. As part of the underlying resistance mechanism to ALK inhibitors, a complex chromoplectic residue forms involving two focal regions on the q-arms of chromosomes 1, 9, and 20 containing the ALK locus following ceritinib treatment, indicating a multifaceted resistance mechanism (11).

Crizotinib is predominantly metabolized by CYP3A4/5 existed in the liver. Hepatotoxicity, as well as interstitial lung disease/pneumonitis, QT interval prolongation, bradycardia, and severe visual loss were previously observed in crizotinib-treated NSCLC cases. Here, the rare incidence of serious or fatal complications such as cellulitis, multiple bone fractures, ARDS or TLS require prompt attention from the treating physicians, albeit with CR of the disease  (10, 19).

**Figure 3:**
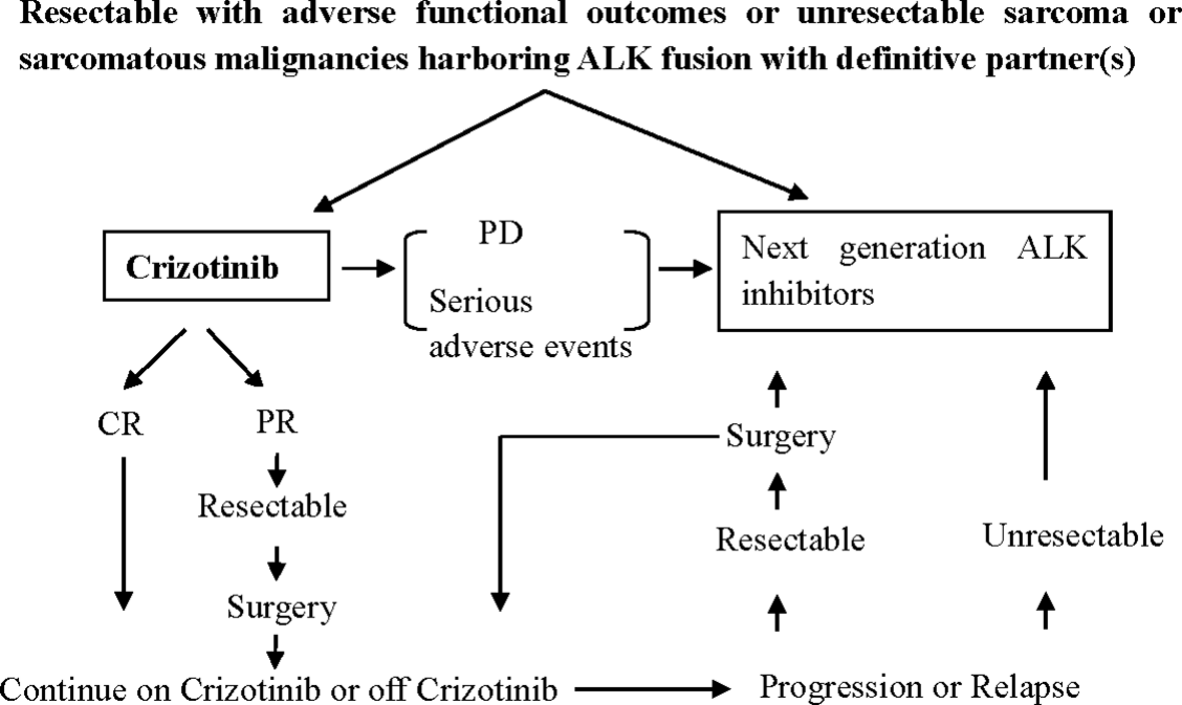
Proposed clinical routine for the treatment of metastatic ALK-positive sarcomatous malignancies. PD, progressive disease; CR, complete remission; PR, partial remission.

This study is not without limitations. Due to the low incidence and high heterogeneity of sarcoma, most of the available data are case reports or subgroup analyses from small size clinical trials. So, the pooled data should be carefully interpreted. Multiple center cooperation for large prospective studies is warranted. A brief clinical routine for treating ALK-positive sarcomatous malignancies was presented in **Figure 3** as a reference for clinical decision-making.

## Conclusion

Findings from this analysis indicated that crizotinib-based palliative treatment regimens improved outcomes in patients with ALK-positive sarcoma and sarcomatoid malignancies. Nevertheless, further investigations are necessary to understand the efficacy of crizotinib treatment over the different types of sarcomas and sarcomatoid malignancies. The potential AEs should be carefully evaluated if crizotinib-based regimens are considered for treatment.

## Author Contributions

Contribution to data retrieval and evaluation: BL and JW collected and entered data. SX, GH and OA reviewed the data. YH and SX, who are sarcoma pathologists, evaluated the outcomes of the information. Contribution to manuscript: BL designed the article. JW drafted the manuscript. YH and GH contributed to the literature search and discussion. OA and SX helped to revise the manuscript. All authors contributed to the article and approved the submitted version.

## Funding

This study was funded by a grant from the Province Natural Science Foundation of Hunan (No. 2021JJ31069).

## Conflict of Interest

The authors declare that the research was conducted in the absence any commercial or financial relationships that could be construed as a potential conflict of interest.

## Publisher’s Note

All claims expressed in this article are solely those of the authors and do not necessarily represent those of their affiliated organizations, or those of the publisher, the editors and the reviewers. Any product that may be evaluated in this article, or claim that may be made by its manufacturer, is not guaranteed or endorsed by the publisher.
